# Validation of a Novel Immunoline Assay for Patient Stratification according to Virulence of the Infecting *Helicobacter pylori* Strain and Eradication Status

**DOI:** 10.1155/2017/8394593

**Published:** 2017-05-30

**Authors:** Luca Formichella, Laura Romberg, Hannelore Meyer, Christian Bolz, Michael Vieth, Michael Geppert, Gereon Göttner, Christina Nölting, Wolfgang Schepp, Arne Schneider, Kurt Ulm, Petra Wolf, Ingrid Lisanne Holster, Ernst J. Kuipers, Bernd Birkner, Erwin Soutschek, Markus Gerhard

**Affiliations:** ^1^Institute for Medical Microbiology, Immunology and Hygiene, Technische Universität München, Munich, Germany; ^2^Department of Pathology, Klinikum Bayreuth, Bayreuth, Germany; ^3^Private Practice for Gastroenterology, Alexanderstr. 2, 95444 Bayreuth, Germany; ^4^Mikrogen GmbH, Neuried, Germany; ^5^Department of Gastroenterology, Hepatology and Gastrointestinal Oncology, Bogenhausen Academic Teaching Hospital, Munich, Germany; ^6^Institute for Medical Statistics and Epidemiology, Technische Universität München, Munich, Germany; ^7^Erasmus MC University Medical Center, Gastroenterology and Hepatology Rotterdam, Rotterdam, Netherlands; ^8^Private Practice for Gastroenterology, Einsteinstraße 1, 81675 Munich, Germany; ^9^DZIF German Centre for Infection Research, Munich, Germany

## Abstract

*Helicobacter pylori* infection shows a worldwide prevalence of around 50%. However, only a minority of infected individuals develop clinical symptoms or diseases. The presence of *H. pylori* virulence factors, such as CagA and VacA, has been associated with disease development, but assessment of virulence factor presence requires gastric biopsies. Here, we evaluate the *H. pylori recom*Line test for risk stratification of infected patients by comparing the test score and immune recognition of type I or type II strains defined by the virulence factors CagA, VacA, GroEL, UreA, HcpC, and gGT with patient's disease status according to histology. Moreover, the immune responses of eradicated individuals from two different populations were analysed. Their immune response frequencies and intensities against all antigens except CagA declined below the detection limit. CagA was particularly long lasting in both independent populations. An isolated CagA band often represents past eradication with a likelihood of 88.7%. In addition, a high *recom*Line score was significantly associated with high-grade gastritis, atrophy, intestinal metaplasia, and gastric cancer. Thus, the *recom*Line is a sensitive and specific noninvasive test for detecting serum responses against *H. pylori* in actively infected and eradicated individuals. Moreover, it allows stratifying patients according to their disease state.

## 1. Introduction

*H. pylori* is a common and widespread bacterial pathogen that infects more than half of the world's population [1]. *H. pylori* colonizes the human stomach and always leads to the development of an active gastritis [2], which in the majority of cases remains asymptomatic. Chronic gastritis may, however, lead to the development of several gastrointestinal diseases such as chronic atrophic gastritis [3], duodenal and gastric ulcers [4], in 1-2% to the development of gastric cancer [5, 6], and lymphoma [7, 8]. Especially, patients with the so-called corpus dominant severe gastritis are at risk for gastric cancer, as severe gastric inflammation can lead to atrophy, metaplasia, dysplasia, and finally, gastric cancer [9]. More than 90% of all gastric cancer cases are associated with a chronic *H. pylori* infection [10] and lead to more than 700,000 stomach cancer-related deaths per year worldwide [11]. Thus, gastric cancer remains the third leading cause of cancer-related mortality [12]. Studies have shown that eradication therapy of *H. pylori* can prevent gastric cancer development [13, 14]. Due to the high number of infected individuals, it is necessary to identify patients at increased risk for gastroduodenal disease in order to subject them to preventive eradication therapy. A number of methods for the detection of *H. pylori* are currently available: histological analysis, microbial culture, and urease breath test are most commonly used. However, the first two diagnostic methods require upper gastrointestinal endoscopy in order to obtain biopsy specimens for testing. None of the aforementioned methods is able to predict the outcome of an *H. pylori* infection in terms of malignant or benign outcome. The line assay system (Helicobacter *recom*Line) analyses immunoglobulin G (IgG) antibody responses to six *H. pylori* antigens (CagA, VacA, GroEL, UreA, HcpC, and gGT), some of which are already known to be linked to a higher risk of disease, as for instance the antigens CagA, VacA, and GroEL [15–17]. The *recom*Line differentiates the more virulent type I *H. pylori* immune response (CagA and/or VacA positive) and the less pathogenic type II immune response (CagA/VacA negative). As the severity of inflammation, and thus immune response, correlates with the risk for gastric cancer [9], the *recom*Line may have predictive properties, which we aimed at evaluating in the present study. We validated the performance of the *recom*Line test compared to histology as a gold standard, with particular focus on the correlation between the *recom*Line test results and chronicity/activity of inflammation as well as most severe lesions according to histology.

As eradication of *H. pylori* has become quite common, tests were developed to confirm treatment success. The Maastricht guidelines consider the urea breath test adequate to confirm successful treatment four weeks after eradication therapy [18]. Antibody responses towards antigens usually sustain for a long period after eradication therapy, as previously shown [19–22]. These studies suggest that serology was not applicable for eradication control. However, while some immune responses to certain antigens persist for decades (especially CagA), antibody titres against other antigens decrease within a shorter period, also depending on the Ig-class tested [23]. This phenomenon could also be utilized as readout to confirm treatment success by analysing the decline of antibody responses, as shown before [24]. Wang et al. conclude from their study that it would be the reasonable and even perhaps preferred method of monitoring *H. pylori* infections [25]. In order to get a better insight into posttreatment IgG immune responses, we analysed sera of a subgroup of patients that had undergone documented eradication therapy by applying the *recom*Line assay.

## 2. Methods

### 2.1. Study Populations and Histology

The analysis was based on a study population recruited between October 2010 and February 2012 by two gastroenterological practitioners in Munich and Bayreuth (Germany). Patients receiving active immunosuppressive therapy, showing coagulation defects, or suffering from malignant diseases were excluded from the study. Serum samples and gastric biopsies of the antrum and corpus were obtained from patients with upper abdominal complaints who underwent gastroscopy after informed consent. The serum samples were stored at −20°C until testing for IgG antibodies against *H. pylori*. For all participants included, age, gender, medical history, and histology status were recorded. *H. pylori* status was defined via histology performed at the Institute of Pathology, Klinikum Bayreuth, or the Institute of Pathology at the Technische Universität München. Therefore, biopsies were fixed in 4% neutral buffered formalin, dehydrated in a series of increasing alcohols and xylene, embedded in paraffin, serially sectioned, deparaffinized, and stained. All biopsies were stained routinely with H&E and immunohistochemical detection of for *H. pylori* at the Institute of Pathology, Klinikum Bayreuth, using Roche monoclonal antibody clone SP48 rabbit-anti-human 790-1014 on Ventana Benchmark Ultra, Strassbourg, France. Pathological evaluation of tissue samples was done according to the updated Sydney System [26]. The chronicity of inflammation was scored by the mucosal infiltration with lymphocytes and plasma cells. The activity of inflammation was scored based on the number of neutrophilic granulocytes within the tunica propria. Both parameters are indicated as mild, moderate, and severe. Apart from chronicity and activity of inflammation, other pathological findings such as atrophy, metaplasia, dysplasia, ulcer, and carcinoma were recorded. For statistical analysis, these were grouped as most severe lesions. No gastric lymphomas were detected in the present study cohort.

Eradicated cases were defined either through histology (Ex-*H. pylori* gastritis) or by documentation of the attending gastroenterologist. The time since eradication was either defined by a known date of eradication (submitted by the gastroenterologist) or determined according to the last date of *H. pylori* positivity in histology, which was followed by eradication therapy and resulted in an Ex-*H. pylori* gastritis in histology or a negative histological result, confirming the success of eradication. In addition, a Dutch cohort was tested prospectively with the *recom*Line not knowing current *H. pylori* status. The study was approved by the ethics committee of the Technische Universität München (May 20, 2009).

### 2.2. The *recom*Line Serologic Assay System

The *recom*Line is based on six recombinant antigens: CagA, VacA, GroEL, UreA, HcpC, and gGT, which are immobilized on a nitrocellulose. On every test strip, a reaction control, antibody control, and cutoff control band are included. For test quantification, the scanner OpticPro S28 (Plustek, Korea) and *recom*Scan software (Mikrogen, Germany) were used according to the manufacturer's instructions. The developed test strips were digitalized by scanning, and the band patterns were assessed via a defined algorithm. The band intensities were determined in more than 750 grey levels and are given in arbitrary units (AU). The recomLine antigens CagA, VacA, and GroEL were scored with two points each, and the other antigens with one point each, adding up to a maximum score of nine points. Results with one point were categorized as borderline and two or more points were considered positive. Representative images of five *recom*Line tests are shown in Supplementary Figure 1 available online at https://doi.org/10.1155/2017/8394593. Detailed information and description on the development of the *recom*Line has been published earlier [27].

### 2.3. Statistical Analysis

Statistical analysis was performed as described before [27]. The performance of the *recom*Line was validated against histological assessment as gold standard calculating the sensitivity and specificity. The immune response frequency was calculated comparing the ratio between patients positive for a certain antigen and the total number of individuals tested positive. The test results for *H. pylori* positive and negative patients analysed with the *recom*Line were validated against histology results. Differences in the study population were assessed using Pearson's *χ*^2^ test (*c*^2^) or the nonparametric Kruskal-Wallis test (*H*). Mean single values were compared using the two-tailed *t*-test with 95% CIs (*t*). Significances in mean band intensity were calculated using the one-way ANOVA (*F*). The *p* values were depicted in the figure legends as follows: letters indicating the test used, number/numbers in parentheses indicating the degree(s) of freedom, and followed by the defined statistics value and the significance level. Differences were rated as significant at *p* < 0.05.

## 3. Results

### 3.1. Patient Population and Performance of *recom*Line Assay

The German cohort of 1291 patients is characterized as follows: 447 (34.6%) patients were *H. pylori* negative and 409 (31.7%) patients were *H. pylori* positive according to histology. The negative population was included to determine the basic test performance values (sensitivity and specificity) [27]. As this manuscript focuses on the serological response of the *H. pylori* positive and eradicated patients, more detailed information on the *H. pylori* negative patients are not presented. 435 (33.7%) patients had previously undergone *H. pylori* eradication treatment. The cumulative (past and present) prevalence of *H. pylori* in the study population was 65.4% and increased with age. *H. pylori*-eradicated patients were significantly (*p* < 0.01) older than *H. pylori* negative patients. The mean age was 53.6 ± 16.2 ranging from 18 to 89 years. In total, more female patients (708, 54.8%) were included in the study. Detailed information on the study populations analysed can be found in the Supplementary Table 1. The performance values previously determined for the *recom*Line assay [27] were confirmed in this extended population, as shown in Supplementary Table 2.

### 3.2. Eradicated Patients Frequently Show Only a CagA Antibody Response

In order to assess (i) how serologic responses to individual antigens changes over time and (ii) if the serological response to individual antigens might have the capacity to differentiate *H. pylori* infection pre- or posttreatment, we investigated the immune response patterns of a subgroup of individuals that had received antibiotic treatment in the past.

**Figure 1:**
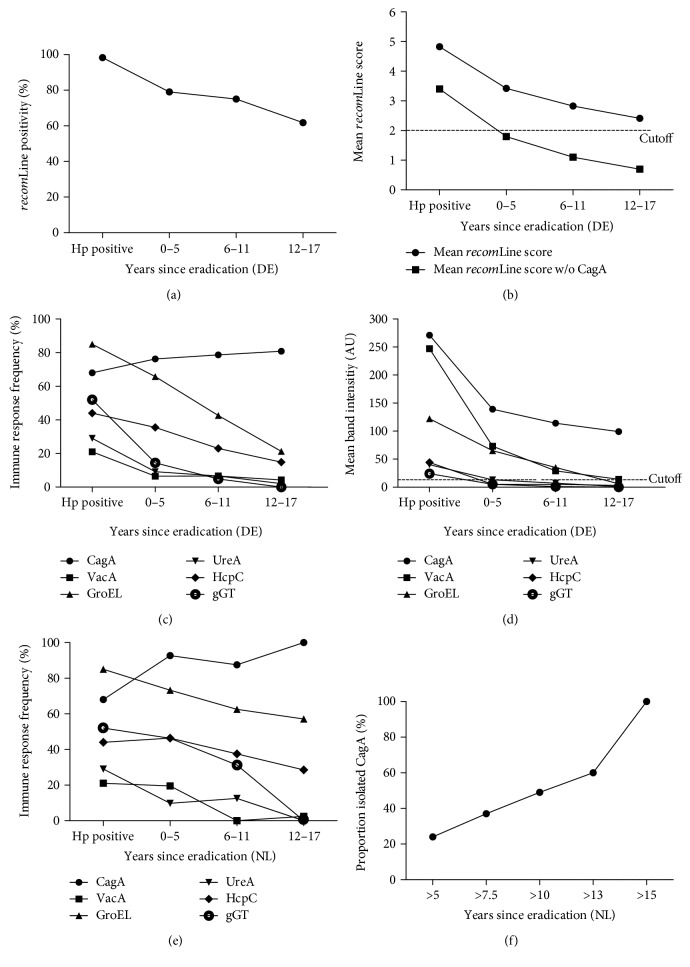
*recom*Line test results from patients without and with eradication therapy. (a) *recom*Line positivity, (b) mean *recom*Line score, (c) immune response frequency, and (d) mean immune response intensity of individuals with an active ongoing *H. pylori* infection (*n*_Hp positive_ = 402) compared to individuals having received antibiotic treatment in the past (*n*_years since eradication_ = 210), indicated as years since eradication and grouped (0–5, 6–11, and 12–17 years) analysing a German cohort (DE). Moreover, the relationship between (e) immune response frequency to individual antigens (●CagA, ■VacA, ▲GroEL, ▼UreA, ♦HcpC, and ○gGT) and (f) percentage of isolated CagA immune response and the time since eradication therapy analysing a Dutch cohort (NL) (*n* = 65). While the immune response for all antigens tested declines after eradication, the response towards CagA antibodies persists over a long period, resulting in an isolated CagA positivity, which remains above cutoff and is significantly increased (*p* < 0.01) in the 12–17 years group compared to all other antigens.

Of all the individuals categorised as eradicated, detailed information on eradication was known for 64.6% (281/435). In total, 74.7% (210/281) of individuals with documented eradication were *recom*Line positive. Moreover, a negative correlation between the time since eradication and a positive test result was observed (Figure 1(a)). The overall *recom*Line positivity rates decreased from 79.0% at 0–5 years after eradication therapy to 61.8% 12–17 years after therapy. This negative correlation could also be observed analysing the mean *recom*Line score (Figure 1(b)).

Besides the decrease of the total number of positive cases, we also analysed the decrease of serologic response on a single antigen level.  Figure 1(c) shows the relationship between the time since eradication therapy and the serological response to the antigens on the *recom*Line compared to individuals with an active ongoing *H. pylori* infection. Noteworthy, the antibody response against all antigens decreased over time except for the CagA response that was particularly long lasting (Supplementary Figure 2). This can be explained by the mean band intensities as shown in Figure 1(d). A decrease in mean serological signal was shown for all antigens tested. However, a significant decrease was demonstrated for CagA (*p* < 0.01), VacA (*p* < 0.01), GroEL (*p* < 0.01), HcpC (*p* < 0.01), and gGT (*p* < 0.01). The mean CagA seroresponse intensity decreased by 43.3% (117/271 AU) over time but compared to the other antigens still showed a significantly higher (*p* < 0.01) mean band intensity above cutoff in the group of 12–17 years after eradication as indicated in Figure 1(d). The signals for the other antigens decrease to or below detection limit six years after eradication, which explains the high isolated CagA positivity rates (only positive for CagA) for this group. Concerning the relative positivity of CagA, we observed that CagA was significantly (*p* < 0.01) more often positive in eradicated patients (86.3%) than in *H. pylori* positive patients with 69.2% positivity (Supplementary Figure 2). Moreover, 43.3% (91/210) of all *recom*Line positive cases in the eradicated cohort showed an isolated CagA seroresponse, compared to 5.5% (22/400) in the group with an ongoing *H. pylori* infection. Comparing these two groups, the probability that a patient showing an isolated CagA band had been treated in the past is 88.7% ((0.433/(0.433 + 0.055))∗100). In order to substantiate this finding, we analysed a Dutch cohort in which the *H. pylori* status was blinded. We initially observed that in this cohort, the mean *recom*Line score was significantly (*t*(595) = 13.0, *p* < 0.001) lower (2.5 ± 2.3) compared to the score of positive cases in the German cohort (4.8 ± 1.9). Upon unblinding, it was confirmed that 84.6% (193/228) of the individuals tested turned out to be *H. pylor*i negative by histology. Analysing the individuals with documented eradication (*n* = 65) in more detail, the antigen frequency in this population showed the same pattern as observed in the German population, defined by markedly lower antibody responses after eradication except for the antibody response towards CagA (Figure 1(e)). As shown in Figure 1(f), depending on the eradication time point, the sensitivity to detect an individual with an isolated CagA immune response in this population increases up to 100% after 15 years. Based on these results, the serological response pattern showing an isolated CagA band might be used to discriminate *H. pylori* positive from eradicated patients when using this serology assay.

### 3.3. *recom*Line Results Correlate with Histological Findings in *H. pylori* Positive Patients

**Figure 2:**
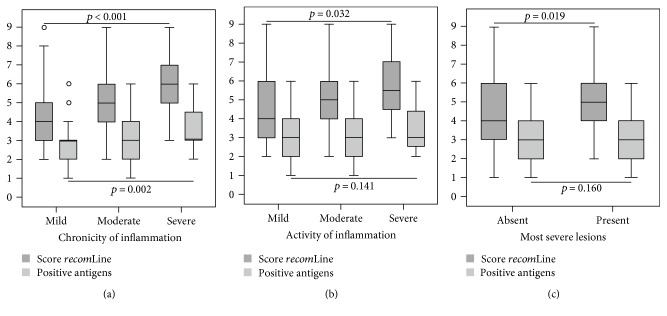
Box plot diagrams showing the association between *recom*Line test results (dark grey: score *recom*Line/light grey: number of positive antigens) and histological findings in *H. pylori* positive cases. (a) Correlation between *recom*Line test results and the chronicity of inflammation (*n* = 391). (b) Correlation according to the activity of inflammation (*n* = 389) as well as (c) pathological findings (*n* = 95) according to histology. Data are presented as box plot showing the median, 75th and 25th percentile, outlier, and extreme values. Significances were calculated using the Kruskal-Wallis test. The results show a significant correlation between the achieved *recom*Line score and histological findings. The sum of positive antigens only correlates significantly with the chronicity of inflammation.

To examine the relationship between the *recom*Line test results and histological findings, we focused on the cases which are positive by histology. Of all the histologically positive cases, 98.3% (400/407) were *recom*Line positive, with the majority of cases being either CagA and/or GroEL positive. Hence, the serological response to the antigens VacA, UreA, and gGT or their combination was important to detect those cases that were CagA and GroEL negative. Moreover, the additional information gained from recognition of these antigens was valuable for the identification of patients with severe chronic and active gastritis as these antigens also have an impact on the overall score. As shown in Figure 2 compared to the number of recognized antigens, the mean *recom*Line score significantly correlates with the chronicity (*p* < 0.01) and activity (*p* = 0.032) of inflammation. Furthermore, the *recom*Line score significantly correlates (*p* = 0.019) with pathologic findings, which were grouped as most severe lesions due to small number of such findings in our population. The mean *recom*Line score was significantly higher (*p* = 0.02) in the group presenting with most severe lesions (5.7 ± 1.8) compared to the mean score of *H. pylori* infected asymptomatic individuals (4.6 ± 1.9).

**Figure 3:**
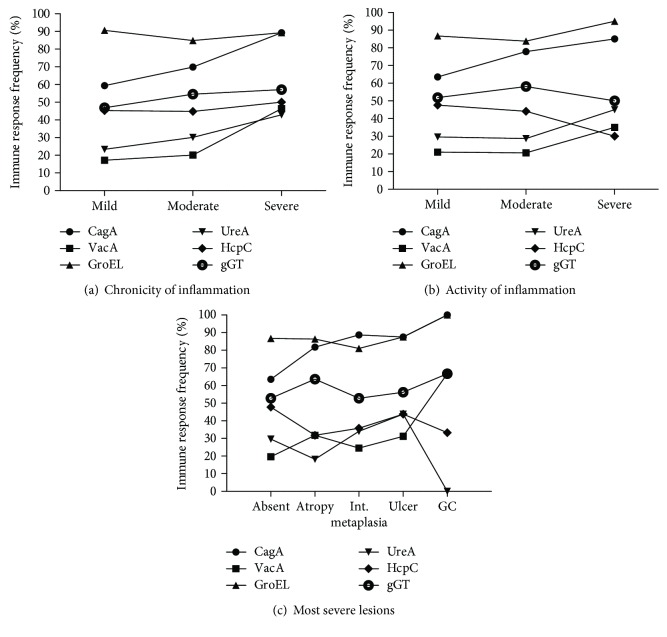
Association between positivity of individual antigens (●CagA, ■VacA, ▲GroEL, ▼UreA, ♦HcpC, and ○gGT) and histologic findings such as (a) chronicity and (b) activity of inflammation as well as (c) most severe lesions. The chronicity and activity are categorized as mild, moderate, and severe. Most severe lesions are categorized as either absent, atrophy, intestinal metaplasia, ulcer, or gastric cancer (GC) according to histology. A significant correlation could be found between CagA or VacA and the chronicity of inflammation (*p* = 0.015; *p* < 0.01, resp.), as well as CagA and the activity of inflammation (*p* < 0.01).

On the level of individual antigens, a significant correlation could be found between CagA or VacA and the chronicity of inflammation (*p* = 0.015 or *p* < 0.01, respectively), as well as CagA and the activity of inflammation (*p* < 0.01) (Figure 3). The serological detection of the antigen UreA tends to increase with disease severity. This, however, was not significant, possibly because of its overall low prevalence in this setup. The antigens GroEL, HcpC, and gGT are stable between the groups analysed (Supplementary Table 3). Next, we looked for correlations between the individual antigens and pathological findings, such as ulcer or atrophy, metaplasia, and carcinoma. A significant correlation could again be seen for CagA (*p* < 0.01). Ulcer and carcinoma cases also showed high seropositivity rates for GroEL with 87.5% and 100%, respectively, but not at a significant correlation because of the high frequency in the control group. A change in seropositivity could also be shown for the antigen VacA, however not at a significant level. Detailed information is given in Supplementary Table 4.

**Figure 4:**
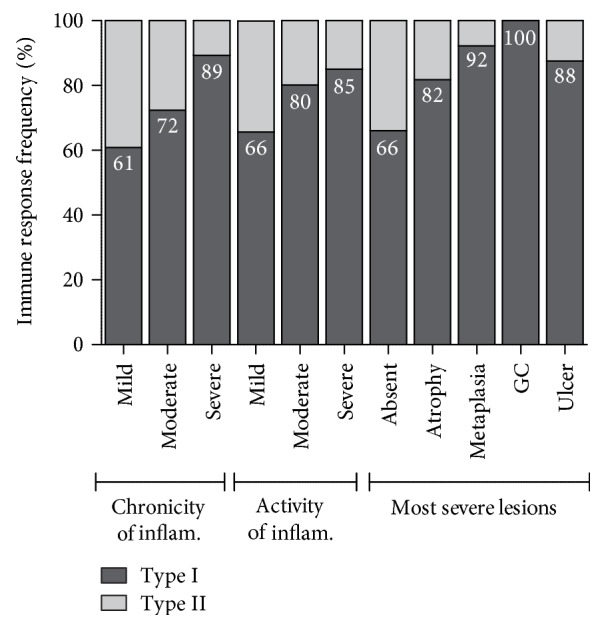
Association between the type of serological response (I/II) and the findings in histology analysing *H. pylori* positive cases. The presence of an immune response against *H. pylori* virulence factors CagA/VacA indicates a type I infection, and these are defined as the high-risk group. The chronicity and activity are categorized as mild, moderate, and severe. Most severe lesions are categorized as absent, atrophy, intestinal metaplasia, ulcer, and gastric cancer according to histology. The proportion of type I immune response increases with higher degrees of chronicity and activity of gastritis (*p* = 0.02 and *p* < 0.01, resp.). There is a significant increase in the portion of type I immune response if signs of advanced pathology are present in histology (*p* < 0.01).

The immune response towards a more virulent *H. pylori* type I was defined by CagA and/or VacA seropositivity and towards the less virulent type II strains by a lack of immune response towards CagA and VacA. Our data show a significant correlation between CagA seropositivity and classification of increasing pathology according to histology. Therefore, a significant correlation was also found for the type of immune response to *H. pylori*. Here, we looked at the 400 cases that were positive for *H. pylori* in histology as well as by *recom*Line. Of these, 71.5% were *H. pylori* type I and 28.5% type II, compared to 87.1% that were classified as type I in the eradicated collective. As shown in Figure 4, the proportion of type I immune response significantly increases with higher scores of chronicity or activity of inflammation (*p* = 0.02 or *p* < 0.01, respectively). Furthermore, there is a significant increase in the share of type I immune response if signs of advanced pathology are present in histology (*p* < 0.01), increasing up to a 100% type I immune response in the gastric cancer group.

## 4. Discussion

*H. pylori* plays an important role in the development of gastroduodenal diseases. Common serological methods for the detection of *H. pylori* do not allow distinguishing between patients with an ongoing *H. pylori* infection and patients that have received eradication therapy in the past. Furthermore, these serological tests are not capable of stratifying patients according to their individual disease state, even though several virulence factors have been known for years. The aim of the present study was to evaluate a new line assay (*recom*Line) with respect to identifying individuals that have received antibiotic treatment. Moreover, we evaluated the capacity of the *recom*Line to stratify patients according to their disease status. The present study confirmed a high sensitivity and specificity of 98.3% and 95.5%, respectively, for the detection of an *H. pylori* infection by the *recom*Line analysing this extended cohort. Nevertheless, serology still plays a minor role in the standard diagnosis of *H. pylori*, even though studies have shown that serological screening for *H. pylori* can reduce the endoscopy workload, which is especially important in countries where access to endoscopy is limited [28].

As our cohort includes a large proportion of individuals that had received antibiotic treatment, we investigated the behaviour of IgG immune responses after successful *H. pylori* eradication in this subpopulation. Current serological tests are incapable of distinguishing an ongoing from a past *H. pylori* infection. Histology as the gold standard in *H. pylori* diagnosis may show an Ex-*H. pylori* gastritis after successful eradication therapy, sometimes even after several decades. However, after due course, the gastritis can also disappear and previous *H. pylori* infection is indeterminable [29]. Furthermore, the urea breath test only allows the identification of an ongoing infection. Thus, it might be helpful to employ the *recom*Line to detect a past *H. pylori* infection. Our results show that a low test score especially in combination with an isolated CagA band may allow the differentiation of *H. pylori* positive and eradicated individuals. The mean band intensity and in parallel the immune response frequency decline after successful *H. pylori* eradication for all antigens tested except for CagA, which was particularly long lasting. Thus, this decline in immune responses has an effect on the test score and the overall *recom*Line positivity. This was corroborated in a prospective study, analysing a blinded Dutch cohort, in which most of the cases turned out to have undergone eradication therapy. Again, the results show a decrease in immune response frequency for all antigens except for CagA. In this cohort, the sensitivity of detecting a status of prior eradication through an isolated CagA band would increase up to 100% after 15 years. This study shows the feasibility of detecting individuals that have received antibiotic treatment by analysing the immune responses to individual antigens. One limitation of our study is that the time span since eradication in most individuals was quite long. For a more precise analysis, a prospective follow-up study with known baseline antigen patterns should be conducted, focusing on shorter time intervals. Furthermore, IgA immune responses should be analysed to draw further conclusions for this antibody class, as this class might be more accurate to detect *H. pylori* pre- and postinfection as suggested by Kato et al. [23].

In addition, we investigated whether or not the detection of immune responses to individual antigens or the overall test score might have the capacity to identify H. pylori positive patients at increased disease risk, if the *recom*Line allows the differentiation between the more virulent type I and the more attenuated type II strains, and if this differentiation might be associated with the disease outcome. The *recom*Line test score, compared to the number of positive antigens, shows a significant correlation with the severity of gastritis (activity and chronicity of inflammation) and most severe lesions. The *recom*Line score was determined by assessing the serological response to six *H. pylori* virulence factors CagA, VacA, GroEL, UreA, HcpC, and gGT. 393 of the 400 *recom*Line positive cases were either CagA and/or GroEL positive. The other four antigens were, thus, needed for detecting those rare cases not being CagA/GroEL positive. Moreover, they were valuable in the calculation of the *recom*Line score, which was employed for the differentiation between *H. pylori* positive and eradicated patients. In addition, we show a significant correlation between the *recom*Line score and the chronicity and activity of inflammation. The more antigens were detected and, thus, the higher the *recom*Line scored, the more likely it was that the respective patient presented with a severe gastritis. The highest *recom*Line scores were found in patients with severe chronic and active gastritis, as well as gastric or duodenal ulcer, and gastric cancer patients. These patients were also more likely to have a more virulent type I *H. pylori* infection. Thus, the *recom*Line test can identify patients at increased risk to present with gastroduodenal disease and allows a further risk stratification concerning the pathogenicity of individual *H. pylori* strains. Thus, the recomLine is the first serologic test with can support risk stratification of *H. pylori* positive patients.

The more virulent *H. pylori* type I strains are in general defined through CagA positivity and secretion of “toxic” VacA s1m1 [30]. Due to the relatively high homology between the different VacA forms and numerous shared epitopes, serology cannot reliably differentiate the respective proteins. A distinction between VacA s1m1 (the more virulent form) and s2m2 (the more attenuated form) is not possible with the current *recom*Line test. However, CagA presence is mostly associated with the more virulent VacA s1m1 form [15]. In total, 285 cases were either CagA or VacA positive, the majority being CagA positive (97.5%). Only 7 cases were VacA positive but CagA negative, which were also calculated as type I immune response. Future *recom*Line optimization should enable the distinction in VacA positivity according to its s and m variants in order to allow a more accurate identification of *H. pylori* type I strains.

Previously, it was reported that antibody responses against GroEL correlate significantly with disease outcome [31]. In our study population, however, seroresponse against GroEL showed no significant correlation with increased pathology, due to its high frequency in the control group. It is known that GroEL has a high homology between *H. pylori* isolates. Therefore, the difference between our findings and published data might be due to antigen production, or the test format applied, which might lead to alterations in epitope recognition.

By identifying patients at increased disease risk, the *recom*Line can help to reduce the number of patients needed to treat and, thus, unnecessary side effects of eradication therapy are avoided. If in our study cohort only patients who carry a more virulent type I *H. pylori* strain (according to serology; *n* = 278) were to be treated in contrast to all *H. pylori* positive patients (*n* = 400), 122 patients (30.5%) could have been spared from probably unnecessary eradication therapy. The 278 cases of type I immune response include the majority of patients showing pathological findings in histology: all (100%) gastric cancer cases, 87.5% (14/16) of the gastric or duodenal ulcer cases (which would also be identified through clinical symptoms), 88.7% (47/53) of the metaplasia cases, 81.8% (18/22) of the atrophy cases, 85.0% (17/20) of the high activity of inflammation, and 89.3% (25/28) of the high chronicity of inflammation cases. Thus, only 18 patients (12.7%) with such findings in histology escaped recognition (10× score ≥ 4; 8× score < 4). If only patients with a type I immune response (*n* = 278) or a score of 4 or higher (*n* = 58, type II immune response) were to be treated, eight cases (5.6%) with pathological findings in histology would not receive treatment (1× ulcer, 4× metaplasia, 1× atrophy, and 2× severe activity of inflammation). However, these patients might be subjected to further endoscopic surveillance due to histological findings. Thus, the *recom*Line supports the identification of patients at higher risk. Nevertheless, histology still remains an important part of standard diagnosis in order to identify all patients with severe gastritis and changes in the gastric mucosa.

Our patients showed a high frequency of type I immune responses in the *H. pylori* positive collective (71.5%). As already mentioned, patients with severe gastritis, metaplasia, ulcer, and carcinoma showed high type I rates. However, patients with already a mild gastritis showed high rates of CagA positivity in both cohorts (61% and 65%, respectively). These patients might be at an early stage of their disease and thus at a milder level of inflammation, as the mean age of these patients (mild chronic and active inflammation and a type I immune response) was 53.9 (±14.6), compared to a mean age of 63.0 (±13.1) in patients with high chronicity and highly active gastritis. Further longitudinal studies are needed in order to assess disease development in such a group compared to a reference group of type II immune response patients in order to validate such prognostic patient stratification.

To further evaluate the predictive properties of the *recom*Line, a larger patient collective has to be screened, as the present cohort included only a minority of advanced stages of gastric disease. Moreover, the inclusion of additional antigens may provide further information concerning the pathogenicity of *H. pylori*. Especially, the discrimination between a type I and type II immune response needs to be further substantiated, for example, by distinguishing seropositivity of the more virulent VacA s1m1 from the s2m2 variant or by identifying new biomarkers.

**Figure 5:**
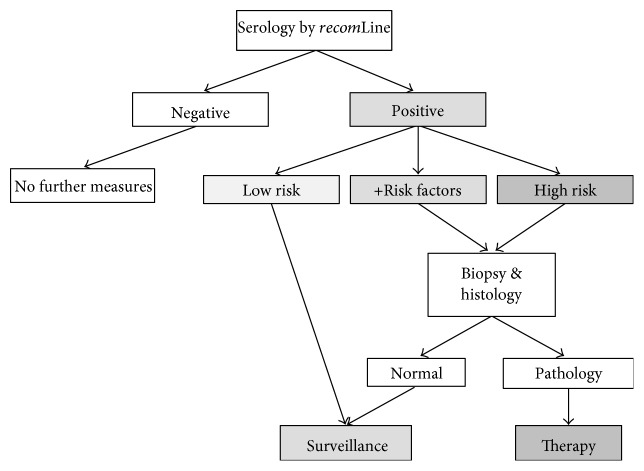
*H. pylori* diagnostic decision tree. Low risk: no seropositivity against CagA and VacA and no additional risk factors; +Risk factors: no seropositivity against CagA and VacA but additional risk factors such as smoking, diet, and genetic predisposition; High risk: seropositivity against CagA and VacA.

We conclude that the *recom*Line is a sensitive, specific, and noninvasive test for the detection of the serum response to an *H. pylori* infection. Moreover, this assay has indicative capacity to identify patients at higher risk for *H. pylori* associated diseases. We, therefore, propose to include *recom*Line-based serology in the clinical handling of *H. pylori* infection according to the decision tree as suggested in Figure 5. We are aware that the practical implementation in the clinical practice presents a challenge for such test. As with many novel diagnostic tools, it may take years to incorporate them into clinical guidelines. Yet, since the first presentation of our data on scientific meetings, several gastroenterologists in Germany have started to use the line blot as an additional means to assess the putative disease risk of *H. pylori*-infected patients presenting with chronic gastritis. Clinical trials investigating the usefulness of such test in a prospective manner (i.e., before endoscopy is performed) are underway and may help to introduce the test into clinical practice. Further, we are currently performing cost-efficiency calculations of the algorithm proposed in Figure 5 to substantiate the advantages of such approach. Thus, the *recom*Line assay could present a valuable tool for an easy-to-perform, cost-effective primary patient screen, and could help to identify patients who require endoscopic evaluation and subsequent antibiotic therapy.

## Supplementary Material

Supplementary Table 1. Study Populations. Supplementary Figure 1. The result of five representative recomLine tests incubated with individual patient sera are shown (LHP13 − LHP17). The assignment of the bands is given in the cartoon above. The test score calculation is given beside the recomLine tests. LHP 16 shows only a CagA band and thus represents a typical test result for an eradicated patient. Supplementary Table 2. Cross Table Histology vs. Serology (recomLine). Supplementary Table 3. Association between gastritis parameters and single H. pylori antigen positivity. Supplementary Table 4. Association between most severe lesions and single antigen H. pylori positivity. Supplementary Figure 2. Antigen frequencies of H. pylori positive and eradicated patients CagA was significantly (p<0.01) more often positive in eradicated patients (86.3%) than in H. pylori positive patients (69.2%), while all other immune responses were lower in the eradicated cohort.
