# Supplementary material for: Validation of a Novel Immunoline Assay for Patient Stratification according to Virulence of the Infecting Helicobacter pylori Strain and Eradication Status
Source: J Immunol Res. 2017 May 30;2017:8394593. doi: 10.1155/2017/8394593 (PMC5468576; doi:10.1155/2017/8394593)
Supplement: Supplementary file 1 — Supplementary Table 1. Study Populations. Supplementary Figure 1. The result of five representative recomLine tests incubated with individual patient sera are shown (LHP13 − LHP17). The assignment of the bands is given in the cartoon above. The test score calculation is given beside the recomLine tests. LHP 16 shows only a CagA band and thus represents a typical test result for an eradicated patient. Supplementary Table 2. Cross Table Histology vs. Serology (recomLine). Supplementary Table 3. Association between gastritis parameters and single H. pylori antigen positivity. Supplementary Table 4. Association between most severe lesions and single antigen H. pylori positivity. Supplementary Figure 2. Antigen frequencies of H. pylori positive and eradicated patients CagA was significantly (p<0.01) more often positive in eradicated patients (86.3%) than in H. pylori positive patients (69.2%), while all other immune responses were lower in the eradicated cohort. [file 8394593.f1.docx]

Supplementary data

**Validation of a novel immuno-line assay for patient stratification according to virulence of the infecting *Helicobacter pylori* strain and eradication status**

*Luca Formichella^1^, Laura Romberg^1^, Hannelore Meyer^1^, Christian Bolz^1^, Michael Vieth^3^, Michael Geppert^2^, Gereon Göttner^4^, Christina Nölting^4^, Wolfgang Schepp^5^, Arne Schneider^5^, Kurt Ulm^6^, Petra Wolf^6^, Ingrid Lisanne Holster^7^, Ernst J. Kuipers^7^, Bernd Birkner^8^, Erwin Soutschek^4^, Markus Gerhard^#^^[[1]](#footnote-1),9^*

|  | Germany (D) | | | | Netherlands (NL) | | | |
| --- | --- | --- | --- | --- | --- | --- | --- | --- |
| histology | positive | negative | eradicated | total | positive | negative | eradicated | total |
| N | 409 | 447 | 435 | 1291 | 35 | 91 | 102 | 228 |
| % | 31.7 | 34.6 | 33.7 | 100 | 15.4 | 39.9 | 44.7 | 100 |
| mean age | 52.7  (± 16.1) | 48.2  (± 16,6) | 59.3  (± 13.7) | 53.6  (± 16.2) | 62.8  (± 12.6) | 61.1  (± 10.6) | 61.5  (± 11.7) | 62.2  (± 11.3) |
| gender (f/m) | 0.53/0.47 | 0.55/0.45 | 0.59/0.41 | 0.55/0.45 | 0.63/0.37 | 0.49/0.51 | 0.40/0.60 | 0.48/0.52 |
| Serology | 409 | 447 | 281 | 1137 | 35 | 91 | 102 | 228 |
| mean recomLine score | 4.8  (± 1.9) | 0.24  (± 0.8) | 3.2  (± 1.8) | N/A^1^ | 5.1  (± 2.1) | 2.7  (± 2.2) | 3.4  (± 2.1) | 2.5  (± 2.3) |
| recomLine + | 400^4^ | 40 | 210^4^ | 650 | 32 | 14 | 65^4^ | 111 |
| % recomLine + | 98.3 | 9.0 | 74.7 | 57.4 | 91.4 | 15.4 | 84.4 | 54.7 |
| recomLine - | 7 | 404 | 71 | 482 | 3 | 77 | 12 | 92 |
| % recomLine - | 1.7 | 91.0 | 25.3 | 42.6 | 8.6 | 84.6 | 15.6 | 45.3 |
| Excluded for analysis | 2^2^ | 3^2^ | 154^3^ | 159 | 35^1^ | 91^1^ | 25^3^ | 151 |

Supplementary Table 1. Study Populations

^1^not applicable; ^2^borderline; ^3^no documentation; ^4^included in analysis


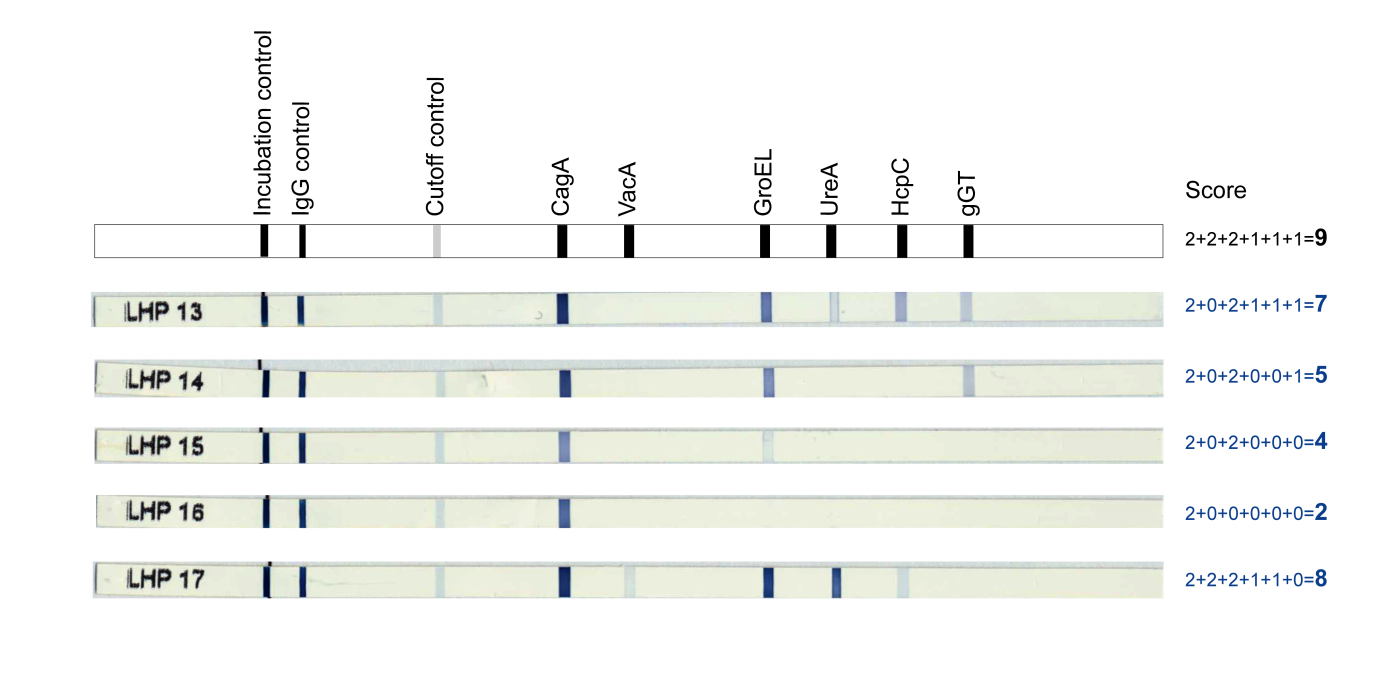


Supplementary Figure 1. The result of five representative *recom*Line tests incubated with individual patient sera are shown (LHP13 – LHP17). The assignment of the bands is given in the cartoon above. The test score calculation is given beside the *recom*Line tests. LHP 16 shows only a CagA band and thus represents a typical test result for an eradicated patient.

|  | | Serology (recomLine) | | total |
| --- | --- | --- | --- | --- |
|  |  | positive | negative |  |
| Histology | positive | 400 | 7 | 407 |
|  | negative | 40* | 404 | 444 |
| total | | 440 | 411 | 851 |

Supplementary Table 2. Cross Table Histology vs. Serology (recomLine)

Sensitivity (recomLine): 98.3%

Specificity (recomLine): 91.0%

*Corrected Specificity (recomLine): 95.5%

(*from 40 false positives, 21 presenting isolated CagA were considered as eradicated)

|  | Chronicity of inflammation | | |  |  |
| --- | --- | --- | --- | --- | --- |
|  | mild | moderate | severe | Total |  |
| N | 64 | 297 | 28 | 389 | p |
| CagA positivity (%) | 59.4 | 70.4 | 89.3 | 69.9 | 0.015 |
| VacA positivity (%) | 17.2 | 20.2 | 46.4 | 21.6 | 0.004 |
| GroEL positivity (%) | 90.6 | 85.5 | 89.3 | 86.6 | 0.506 |
| UreA positivity (%) | 23.4 | 30.3 | 42.9 | 30.1 | 0.173 |
| HcpC positivity (%) | 45.3 | 44.4 | 50.0 | 45.0 | 0.851 |
| gGT positivity (%) | 46.9 | 54.9 | 57.1 | 53.7 | 0.473 |

|  | Activity of inflammation | | |  |  |
| --- | --- | --- | --- | --- | --- |
|  | mild | moderate | severe | Total |  |
| N | 229 | 138 | 20 | 387 | p |
| CagA positivity (%) | 63.8 | 78.3 | 85.0 | 70.0 | 0.0044 |
| VacA positivity (%) | 20.5 | 21.7 | 35.0 | 21.7 | 0.3226 |
| GroEL positivity (%) | 87.3 | 84.1 | 95.0 | 86.6 | 0.3533 |
| UreA positivity (%) | 29.7 | 29.0 | 45.0 | 30.2 | 0.3336 |
| HcpC positivity (%) | 46.7 | 44.9 | 30.0 | 45.2 | 0.3536 |
| gGT positivity (%) | 52.0 | 58.7 | 50.0 | 54.3 | 0.4228 |

Supplementary Table 3. Association between gastritis parameters and single *H. pylori* antigen positivity

|  | Signs of pathology | | | | |  |  |
| --- | --- | --- | --- | --- | --- | --- | --- |
|  | absent | atrophy | metaplasia | ulcer | GC | Total |  |
| N | 299 | 22 | 53 | 16 | 3 | 393 | p |
| CagA positivity (%) | 63.9 | 81.8 | 88.7 | 87.5 | 100.0 | 69.5 | 0.001 |
| VacA positivity (%) | 19.7 | 31.8 | 24.5 | 31.3 | 66.7 | 21.9 | 0.157 |
| GroEL positivity (%) | 87.3 | 86.4 | 81.1 | 87.5 | 100.0 | 86.5 | 0.746 |
| UreA positivity (%) | 29.8 | 18.2 | 34.0 | 43.8 | 0.0 | 30.0 | 0.333 |
| HcpC positivity (%) | 47.5 | 31.8 | 35.8 | 43.8 | 33.3 | 44.8 | 0.373 |
| gGT positivity (%) | 53.2 | 63.6 | 52.8 | 56.3 | 66.7 | 53.9 | 0.885 |

Supplementary Table 4. Association between most severe lesions and single antigen *H. pylori* positivity

Supplementary Figure 2. Antigen frequencies of *H. pylori* positive and eradicated patients CagA was significantly (p<0.01) more often positive in eradicated patients (86.3%) than in H. pylori positive patients (69.2%), while all other immune responses were lower in the eradicated cohort

1. [↑](#footnote-ref-1)
