# Supplementary material for: Validation of a Novel Immunoline Assay for Patient Stratification according to Virulence of the Infecting Helicobacter pylori Strain and Eradication Status
Source: J Immunol Res. 2017 May 30;2017:8394593. doi: 10.1155/2017/8394593 (PMC5468576; doi:10.1155/2017/8394593)
Supplement: Supplementary file 2 [file 8394593.f2.pdf]

Supplementary data

Validation of a novel immuno-line assay for patient stratification according to virulence of the infecting *Helicobacter pylori* strain and eradication status

Luca Formichella[1], Laura Romberg[1], Hannelore Meyer[1], Christian Bolz[1], Michael Vieth[3], Michael Geppert[2], Gereon Göttner[4], Christina Nölting[4], Wolfgang Schepp[5], Arne Schneider[5], Kurt Ulm[6], Petra Wolf[6], Ingrid Lisanne Holster[7], Ernst J. Kuipers[7], Bernd Birkner[8], Erwin Soutschek[4], Markus Gerhard[#1,9]

	GERMANY (D)				NETHERLANDS (NL)			
**Histology**	positive	negative	eradicated	total	positive	negative	eradicated	total
**N**	409	447	435	1291	35	91	102	228
**%**	31.7	34.6	33.7	100	15.4	39.9	44.7	100
**mean age**	52.7 (± 16.1)	48.2 (± 16,6)	59.3 (± 13.7)	53.6 (± 16.2)	62.8 (± 12.6)	61.1 (± 10.6)	61.5 (± 11.7)	62.2 (± 11.3)
**Gender (f/m)**	0.53/0.47	0.55/0.45	0.59/0.41	0.55/0.45	0.63/0.37	0.49/0.51	0.40/0.60	0.48/0.52
**Serology**	409	447	281	1137	35	91	102	228
**mean recomLine score**	4.8 (± 1.9)	0.24 (± 0.8)	3.2 (± 1.8)	N/A[1]	5.1 (± 2.1)	2.7 (± 2.2)	3.4 (± 2.1)	2.5 (± 2.3)
**recomLine +**	400[4]	40	210[4]	650	32	14	65[4]	111
**% recomLine +**	98.3	9.0	74.7	57.4	91.4	15.4	84.4	54.7
**recomLine -**	7	404	71	482	3	77	12	92
**% recomLine -**	1.7	91.0	25.3	42.6	8.6	84.6	15.6	45.3
**Excluded for analysis**	2[2]	3[2]	154[3]	159	35[1]	91[1]	25[3]	151

Supplementary Table 1. Study Populations

[1]not applicable; [2]borderline; [3]no documentation; [4]included in analysis

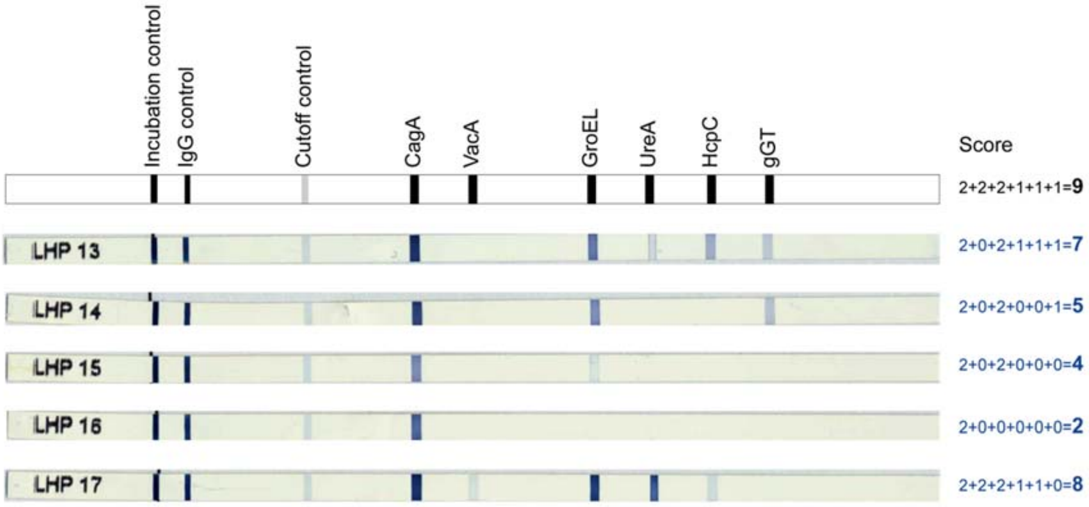

Supplementary Figure 1. The result of five representative *recom*Line tests incubated with individual patient sera are shown (LHP13 – LHP17). The assignment of the bands is given in the cartoon above. The test score calculation is given beside the *recom*Line tests. LHP 16 shows only a CagA band and thus represents a typical test result for an eradicated patient.

		Serology (recomLine)		total
		positive	negative	
Histology	positive	400	7	407
	negative	40*	404	444
total		440	411	851

Supplementary Table 2. Cross Table Histology vs. Serology (recomLine)

Sensitivity (recomLine): 98.3%

Specificity (recomLine): 91.0%

*Corrected Specificity (recomLine): 95.5%

(*from 40 false positives, 21 presenting isolated CagA were considered as eradicated)

Chronicity of inflammation

	mild	moderate	severe	Total	
**N**	64	297	28	389	p
**CagA positivity (%)**	59.4	70.4	89.3	69.9	0.015
**VacA positivity (%)**	17.2	20.2	46.4	21.6	0.004
**GroEL positivity (%)**	90.6	85.5	89.3	86.6	0.506
**UreA positivity (%)**	23.4	30.3	42.9	30.1	0.173
**HcpC positivity (%)**	45.3	44.4	50.0	45.0	0.851
**gGT positivity (%)**	46.9	54.9	57.1	53.7	0.473

Activity of inflammation

	mild	moderate	severe	Total	
**N**	229	138	20	387	p
**CagA positivity (%)**	63.8	78.3	85.0	70.0	0.0044
**VacA positivity (%)**	20.5	21.7	35.0	21.7	0.3226
**GroEL positivity (%)**	87.3	84.1	95.0	86.6	0.3533
**UreA positivity (%)**	29.7	29.0	45.0	30.2	0.3336
**HcpC positivity (%)**	46.7	44.9	30.0	45.2	0.3536
**gGT positivity (%)**	52.0	58.7	50.0	54.3	0.4228

Supplementary Table 3. Association between gastritis parameters and single *H. pylori* antigen positivity

	Signs of pathology						
	absent	atrophy	metaplasia	ulcer	GC	Total	
**N**	299	22	53	16	3	393	p
**CagA positivity (%)**	63.9	81.8	88.7	87.5	100.0	69.5	0.001
**VacA positivity (%)**	19.7	31.8	24.5	31.3	66.7	21.9	0.157
**GroEL positivity (%)**	87.3	86.4	81.1	87.5	100.0	86.5	0.746
**UreA positivity (%)**	29.8	18.2	34.0	43.8	0.0	30.0	0.333
**HcpC positivity (%)**	47.5	31.8	35.8	43.8	33.3	44.8	0.373
**gGT positivity (%)**	53.2	63.6	52.8	56.3	66.7	53.9	0.885

Supplementary Table 4. Association between most severe lesions and single antigen *H. pylori* positivity

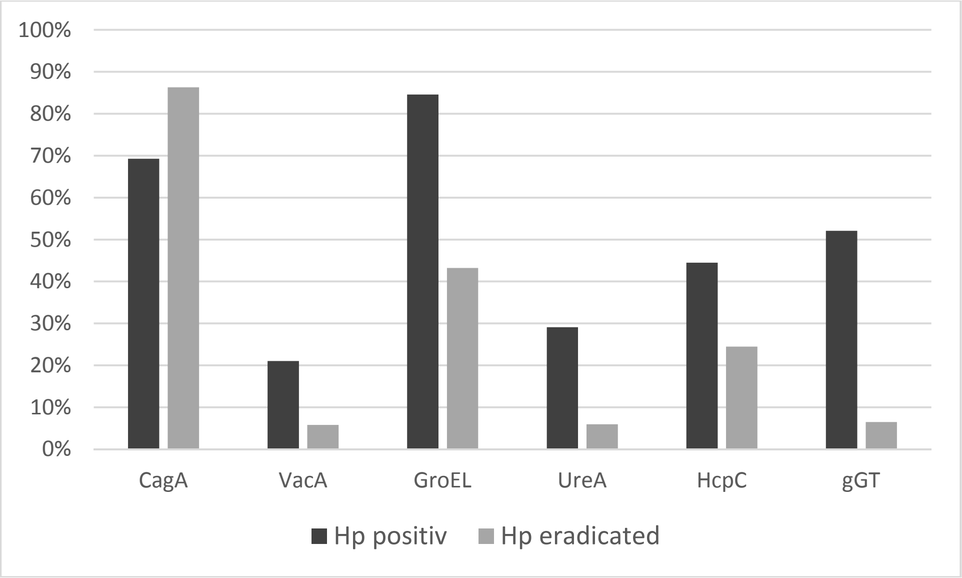

Supplementary Figure 2. Antigen frequencies of *H. pylori* positive and eradicated patients
CagA was significantly ($p<0.01$) more often positive in eradicated patients (86.3%) than in H. pylori positive patients (69.2%), while all other immune responses were lower in the eradicated cohort
